# Dupilumab Improves Endoscopic Features in Adult and Adolescent Patients with Eosinophilic Esophagitis: Post Hoc Analysis of LIBERTY EoE TREET Study

**DOI:** 10.14309/ctg.0000000000001090

**Published:** 2026-08-27

**Authors:** Evan S. Dellon, Margaret H. Collins, Kathryn Peterson, Xian Sun, Bram P. Raphael, Margee Louisias, Allen Radin

**Affiliations:** 1 Center for Esophageal Diseases and Swallowing, Division of Gastroenterology and Hepatology, University of North Carolina School of Medicine, Chapel Hill, North Carolina, USA;; 2 Division of Pathology and Laboratory Medicine, Department of Pediatrics, Cincinnati Children’s Hospital Medical Center, Department of Pathology & Laboratory Medicine, University of Cincinnati College of Medicine, Cincinnati, Ohio, USA;; 3 Division of Gastroenterology, University of Utah, Salt Lake City, Utah, USA;; 4 Regeneron Pharmaceuticals Inc., Tarrytown, New York, USA;; 5 Sanofi, Cambridge, Massachusetts, USA.

**Keywords:** endoscopy, esophageal strictures, eosinophilic esophagitis, inflammation

## Abstract

**INTRODUCTION:**

Endoscopic features are critical for monitoring treatment response in eosinophilic esophagitis (EoE). This post hoc analysis assessed dupilumab's efficacy on endoscopic features of EoE in adults and adolescents in the phase 3 LIBERTY EoE TREET study (NCT03633617).

**METHODS:**

Data were pooled from 2 study arms in which patients with active EoE were randomized to dupilumab 300 mg weekly or placebo for 24 weeks (Parts A/B), followed by dupilumab 300 mg weekly to week (W) 52 (Parts A–C/B–C). EoE Endoscopic Reference Score (EREFS) and subscores (inflammatory and fibrostenotic) were calculated at baseline, W24, and W52 based on worst observed region (WOR), the approach recommended for clinical settings.

**RESULTS:**

At W24, patients showed greater improvement with dupilumab vs placebo in EREFS WOR total score (least squares mean difference [95% confidence interval] −2.17 [–2.59, −1.75], inflammatory subscore −1.65 [–1.96, −1.35], and fibrostenotic subscore −0.50 [–0.71, −0.29], all *P* < 0.0001) and individual EREFS WOR components. The proportions of patients achieving endoscopic remission (≤2) and endoscopic normalization (0) in EREFS WOR total score, and endoscopic normalization in inflammatory and fibrostenotic subscores, were greater with dupilumab vs placebo. Improvements were maintained to W52. Dupilumab showed improvements in EREFS WOR scores vs placebo across endoscopic phenotypes (inflammatory and mixed inflammatory/fibrostenotic).

**DISCUSSION:**

Dupilumab improved endoscopic inflammatory and fibrostenotic features of EoE and led to the normalization of endoscopic features compared with placebo at W24. Improvements with dupilumab were sustained to W52.

## Graphical abstract

**Figure:**
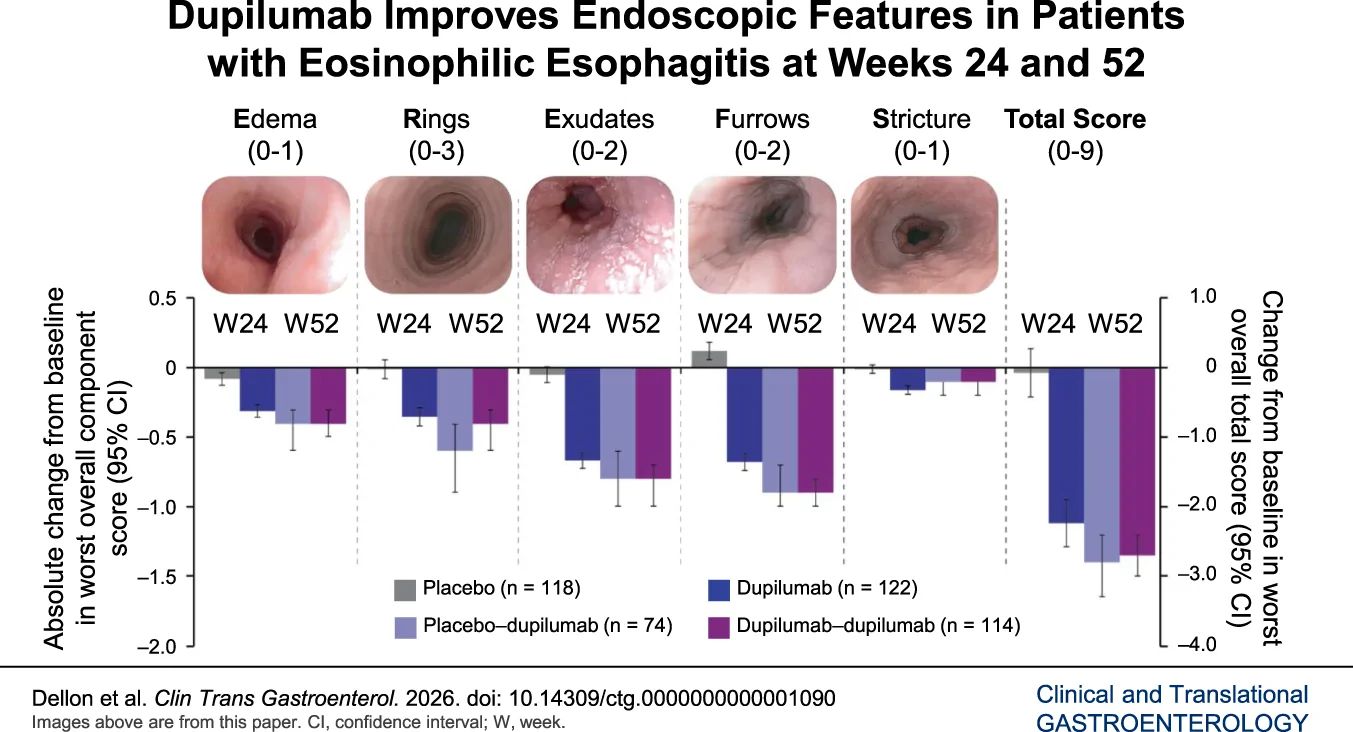

## INTRODUCTION

Eosinophilic esophagitis (EoE) is a chronic, progressive, type 2 inflammatory disease with increasing incidence and prevalence in children and adults (1,2). The diagnosis of EoE is based on the presence of symptoms related to esophageal dysfunction, such as dysphagia, and findings of ≥15 eosinophils per high-power field in esophageal mucosa on biopsy, after evaluating for non-EoE disorders (3). If left untreated, complications such as strictures may occur (4). Esophageal endoscopy is therefore essential for the diagnosis and long-term monitoring of EoE, both to obtain mucosal biopsies for histology (even if the mucosa appears normal) and for endoscopic assessment of esophageal features that are highly supportive of the diagnosis and keys to ongoing monitoring of treatment response (3).

The EoE Endoscopic Reference Score (EREFS) is an objective, standardized, and validated assessment of the severity of the 5 most common endoscopic features in patients with EoE: edema, rings, exudates, furrows, and strictures (5). Individual components can be further classified according to inflammatory (edema, exudates, and furrows) and fibrotic (rings and strictures) features (6). The EREFS is reliable, with substantial inter-rater and intrarater agreement (6), and offers real-time clinical insights beyond symptomatic outcomes and histology, providing a comprehensive measure of endoscopic disease activity in EoE (4,5,7–10). EREFS has been shown to be a sensitive measure of responsiveness in clinical trials of swallowed topical corticosteroids and food-elimination diets, based on histologic analyses (8,11), and endoscopic normalization as assessed by EREFS has been linked to a need for fewer future esophageal dilations (12). Furthermore, the 2025 American College of Gastroenterology guidelines for the diagnosis and management of EoE, as well as the 2022 American Society for Gastrointestinal Endoscopy consensus conference, recommended the use of EREFS to assess endoscopic findings of EoE (3,13). These recommendations, together with recent clinical research demonstrating the limitations of using peak esophageal intraepithelial eosinophil count as the only marker of treatment response in EoE (14–17), suggest that EREFS should be included alongside symptomatic and histologic assessments to provide a comprehensive picture of EoE disease activity.

Dupilumab is a fully human monoclonal antibody that blocks the interleukin-4 and interleukin-13 pathways and has US Food and Drug Administration approvals in multiple diseases marked by type 2 inflammation, including EoE (18,19). The LIBERTY EoE TREET (NCT03633617) was a 3-part, double-blind, placebo-controlled, phase 3 study in which dupilumab 300 mg weekly (qw) demonstrated improvements from baseline vs placebo in the total EREFS score (calculated as the sum of the proximal and distal findings), histologic, symptomatic, and molecular features of EoE at week (W) 24, with improvements maintained at W52 (20,21).

In this post hoc analysis of the data from pivotal dupilumab studies in adolescents and adults, we investigated the effect of dupilumab treatment on these specific endoscopic findings because they are outcomes directly followed in clinical practice.

## METHODS

### Study population

The LIBERTY EoE TREET study design and population have been described previously (20). In brief, eligible patients were aged 12 years or older, weighed ≥40 kg, and had a documented diagnosis of EoE by endoscopic biopsy (peak esophageal intraepithelial eosinophil count ≥15 eosinophils per high-power field) from at least 1 esophageal region despite 8 weeks of high-dose proton-pump inhibitor therapy, a Dysphagia Symptom Questionnaire score of ≥10 at baseline, and ≥4 episodes of dysphagia in 2 weeks. Patients with grade 3 rings or any esophageal stricture unable to be passed by a standard endoscope (<10 mm), or requiring dilation, or who had undergone prior esophageal surgery, were excluded. This analysis presents pooled data from all patients who were randomized to receive dupilumab 300 mg qw or placebo in Parts A and B and received dupilumab 300 mg qw in Parts A–C and B–C.

### Assessments

Endoscopy was performed and EREFS based on worst observed regions (EREFS WOR) at baseline, W24, and W52 was scored locally for Parts A and A–C, whereas Parts B and B–C used central reading if available; otherwise, local reading was used (5,6). Endoscopists scored proximal and distal esophageal regions separately using the furrows-expanded EREFS WOR scoring scheme (6). EREFS WOR total score and inflammatory and fibrostenotic subscores were calculated using the following ranges: EREFS total score 0–9, inflammatory subscore 0–5, and fibrostenotic subscore 0–4. Details of the scoring system, including ranges for EREFS component scores and subscores, as well as details on the categorization of patients into inflammatory and mixed phenotypes, are provided in the Supplementary Methods (see Supplementary Digital Content, https://links.lww.com/CTG/B588). In addition to the assessment of EREFSs, patients' response to treatment was assessed using EREFS WOR total score response thresholds of ≤2 and 0 (10).

Based on baseline EREFS component scores (12), patients were classified as having an inflammatory, mixed inflammatory/fibrostenotic, or fibrostenotic-only phenotype (see Supplementary Table S1, Supplementary Digital Content, https://links.lww.com/CTG/B588). Analyses focused on inflammatory and mixed phenotypes; fibrostenotic-only patients were excluded because of small numbers.

### Statistical analysis

Scores were reported as mean (SD), least squares (LS) mean change, mean change, or percent change (all with 95% confidence interval [CI]). *P* values were calculated using the Cochran-Mantel-Haenszel test for binary variables and analysis of covariance for continuous variables, adjusted for age group and proton-pump inhibitor use. Outcomes were exploratory/post hoc with nominal *P* values. Outcomes in Part C were summarized descriptively without hypothesis testing.

For the absolute or percent change EREFS WOR, values after the first rescue treatment use were set to missing (censored); missing values were imputed by worst observation carried forward method if not due to COVID-19 and by multiple imputations if due to COVID-19. For the EREFS WOR-threshold analyses, values after the first rescue treatment were also censored; however, patients with missing values at each visit were considered nonresponders.

Univariate linear regression analyses were performed to identify baseline characteristics potentially associated with improvements in EREFS WOR total score at W24. The baseline covariates analyzed were weight >60 kg, body mass index (BMI) ≥25–30, BMI ≥30, duration of EoE ≥5 years, history of atopic dermatitis, history of food-elimination diet, history of proton-pump–inhibitor treatment, mixed inflammatory profile on EREFS WOR, and inflammatory profile on EREFS WOR.

## RESULTS

A total of 240 patients were included in this analysis: 118 patients who received placebo and 122 who received dupilumab 300 mg qw in Parts A and B (Figure 1). Baseline demographics and characteristics were similar in the placebo and dupilumab groups (Table 1). At baseline, 0.8% (1/118) and 2.5% (3/122) of patients in the dupilumab and placebo groups, respectively, had EREFS WOR total score 0; 14.4% (17/118) and 11.7% (14/120), respectively, had EREFS WOR total score ≤2.

**Figure 1.**
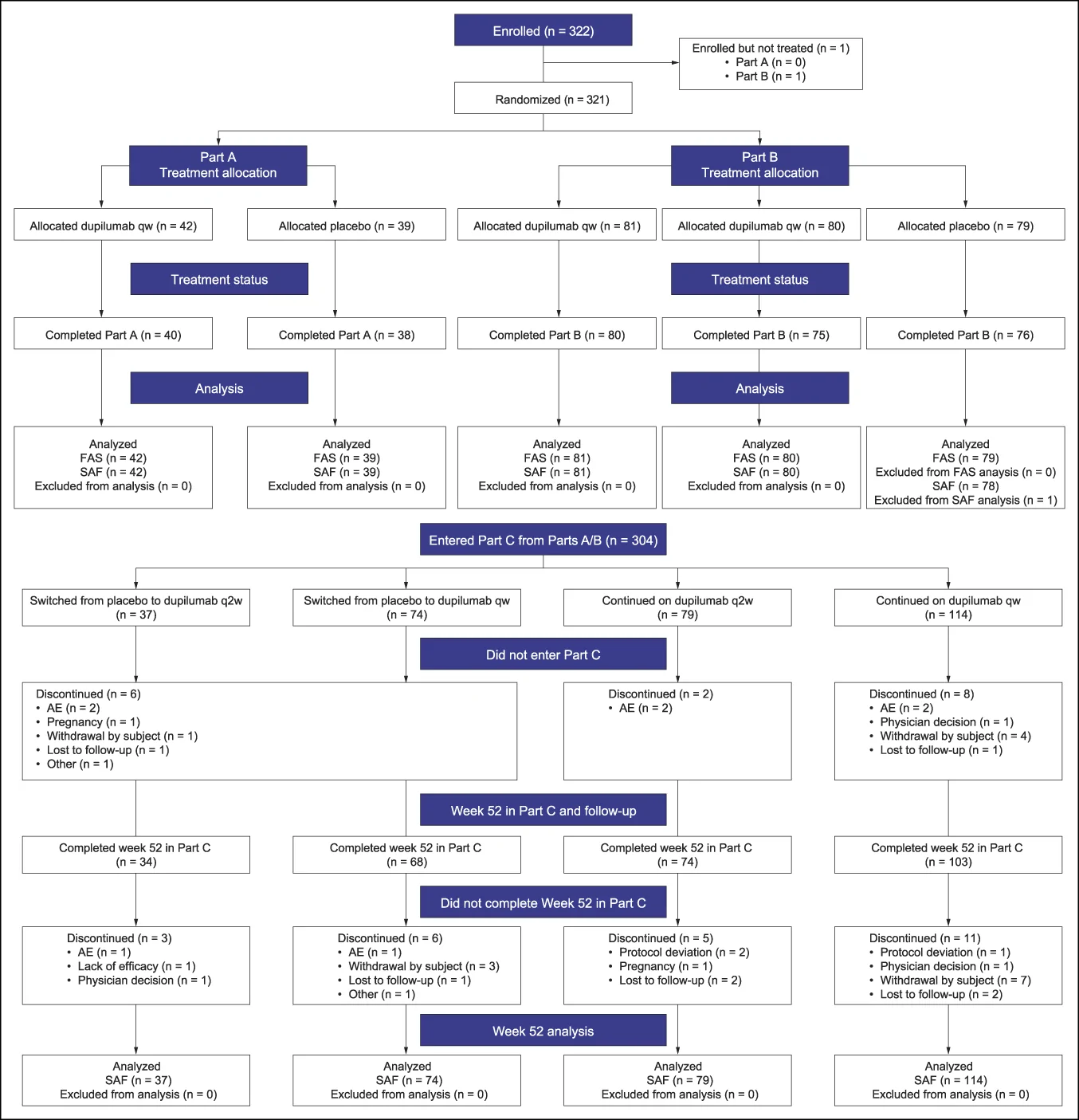
CONSORT diagram. AE, adverse event; FAS, full analysis set; q2w, once every 2 weeks; qw, weekly; SAF, safety analysis set.

**Table 1.** Baseline demographics and disease characteristics of patients who were randomized to receive placebo or dupilumab 300 mg qw in Parts A and B of the LIBERTY EoE TREET

Characteristic	Placebo (n = 118)	Dupilumab (n = 122)
Age, yr, mean (SD)	28.2 (12.5)	30.5 (14.5)
Sex, female, n (%)	39 (33.1)	44 (36.1)
Ethnicity, n (%)		
Hispanic or Latino	6 (5.1)	9 (7.4)
Other	112 (94.9)	113 (92.6)
Race, n (%)		
White	109 (92.4)	112 (91.8)
Black or African American	4 (3.4)	3 (2.5)
Asian	1 (0.8)	3 (2.5)
Other	3 (2.5)	3 (2.5)
Not reported	1 (0.8)	1 (0.8)
History of previously swallowed topical corticosteroids	87 (73.7)	94 (77.0)
PPI use at randomization, n (%)^a^	86 (72.9)	86 (70.5)
On food-elimination diet at screening, n (%)	45 (38.1)	48 (39.3)
Duration of EoE, yr, mean (SD)	4.8 (4.5)	5.7 (4.5)
EREFS WOR total score, mean (SD)	4.0 (1.6)	4.0 (1.6)
EREFS WOR total score 0, n (%)	1 (0.8)	3 (2.5)
EREFS WOR total score ≤2, n (%)	17 (14.4)	14 (11.7)
Individual EREFS WOR scores, mean (SD)		
Edema	0.9 (0.3)	0.8 (0.4)
Rings	1.0 (0.9)	0.9 (0.8)
Exudates	0.9 (0.7)	0.9 (0.8)
Furrows	1.1 (0.6)	1.2 (0.5)
Strictures	0.2 (0.4)	0.2 (0.4)
EREFS WOR inflammatory subscore, mean (SD)	2.9 (1.2)	2.9 (1.2)
EREFS WOR fibrotic subscore, mean (SD)	1.2 (1.1)	1.1 (1.1)
PEC in 3 regions, eos/hpf, mean (SD)	88.3 (46.2)	86.9 (44.8)
DSQ score, mean (SD)	35.8 (11.1)	36.3 (11.7)
History of ≥1 atopic condition, n (%)^b^	104 (88.1)	104 (85.2)

DSQ, Dysphagia Symptom Questionnaire; EoE, eosinophilic esophagitis; eos/hpf, eosinophils per high-power field; EREFS WOR, EoE endoscopic reference score based on worst observed regions; PEC, peak esophageal intraepithelial eosinophil count; PPI, proton-pump inhibitor; qw, weekly.

a Per interactive web response system.

b Medical history recorded of a type 2 inflammatory disease such as asthma, atopic dermatitis, food allergy, or allergic rhinitis.

At W24, dupilumab reduced EREFS WOR total score vs placebo (LS mean change from baseline [95% CI]: −2.24 [–2.58, −1.90] vs −0.07 [–0.42, 0.28]; LS mean difference [95% CI]: −2.17 [–2.59, −1.75], *P* < 0.0001) (Figure 2). Improvements were sustained at W52 with continuous dupilumab treatment (mean change from baseline [95% CI]: −2.7 [–3.0, −2.4]), and improvements were observed in patients who switched to dupilumab at W24 (mean change from baseline [95% CI]: −2.8 [–3.3, −2.4]). Dupilumab improved individual EREFS WOR components vs placebo at W24. At W52, improvements were maintained in patients who received continuous dupilumab, and improvements were seen in patients who switched to dupilumab at W24.

**Figure 2.**
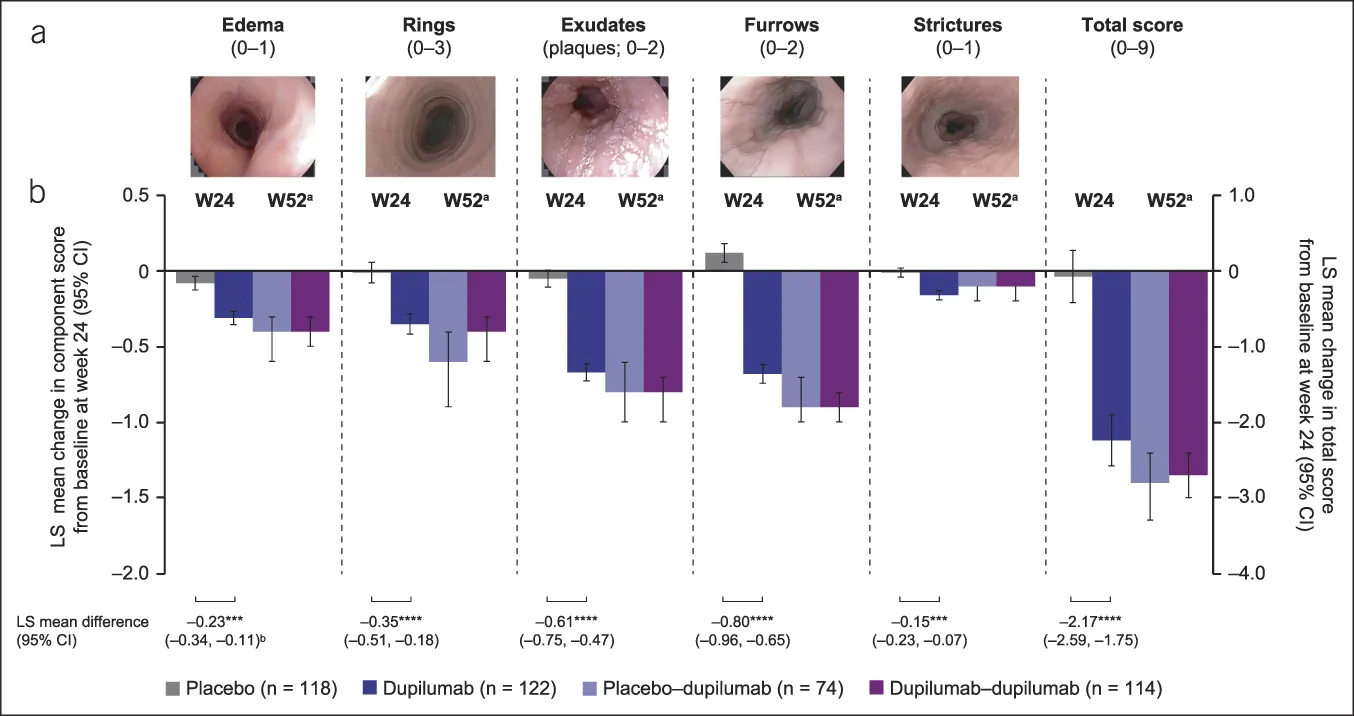
(**a**) Representative EREFS component endoscopy images and (**b**) absolute mean change from baseline in the EREFS total and component scores by worst observed region at W24 and W52. For (**a**), the images were cropped to highlight areas of interest, and the brightness, contrast, and white balance were adjusted. ***P < 0.001. ****P < 0.0001. CI, confidence interval; EREFS, Eosinophilic Esophagitis Endoscopic Reference Score; LS, least squares; W, week. ^a^W52 is the mean change from baseline.

Dupilumab treatment reduced EREFS WOR inflammatory subscore vs placebo at W24 (LS mean change from baseline [95% CI]: −1.67 [–1.92, −1.42] vs −0.01 [–0.26, 0.24]; LS mean difference [95% CI]: −1.65 [–1.96, −1.35], *P* < 0.0001). At W52, benefits were sustained with continuous dupilumab (mean change from baseline [95% CI]: −2.2 [–2.4, −1.9]), and improvements were observed in patients who switched to dupilumab at W24 (mean change from baseline [95% CI]: −2.1 [–2.4, −1.7]). Similarly, dupilumab reduced fibrostenotic subscore vs placebo at W24 (LS mean change from baseline [95% CI]: −0.51 [–0.68, −0.34] vs −0.01 [–0.19, 0.16]; LS mean difference [95% CI]: −0.50 [–0.71, −0.29], *P* < 0.0001). At W52, benefits were sustained with continuous dupilumab (mean change from baseline [95% CI]: −0.6 [–0.7, −0.4]) and improvements were observed in patients who switched to dupilumab at W24 (mean change from baseline [95% CI]: −0.8 [–1.0, −0.5]).

The inflammatory phenotype and mixed inflammatory/fibrostenotic groups had a mean (SD) age of 20.7 (10.9) and 33.8 (12.4) years, respectively. Twelve (15.2%) patients with an inflammatory phenotype had a history of esophageal dilation vs 79 (52.0%) patients with a mixed phenotype. The mean (SD) disease duration was 5.2 (4.6) and 5.3 (4.5) years in the inflammatory phenotype vs mixed phenotype groups, respectively. A greater proportion of patients with inflammatory phenotype had a history of allergic rhinitis, food allergy, and asthma than patients with a mixed phenotype (see Supplementary Table S2, Supplementary Digital Content, https://links.lww.com/CTG/B588). Baseline disease demographics and characteristics were otherwise similar in the inflammatory and mixed phenotype groups.

At W24, dupilumab improved EREFS WOR total scores vs placebo across endoscopic inflammatory phenotype (LS mean change from baseline [95% CI]: −1.74 [–2.26, −1.23] vs 0.22 [–0.28, 0.71]; LS mean difference [95% CI]: −1.96 [–2.62, −1.30]; *P* < 0.0001) and mixed phenotype (LS mean change from baseline [95% CI]: −2.63 [–3.14, −2.12] vs −0.34 [–0.82, 0.15]; LS mean difference [95% CI]: −2.29 [–2.84, −1.74]; *P* < 0.0001). Inflammatory subscore also improved with dupilumab vs placebo across inflammatory phenotype (LS mean change from baseline [95% CI]: −1.90 [–2.34, −1.45] vs −0.16 [–0.60, 0.27]; LS mean difference [95% CI]: 1.73 [–2.31, −1.16]; *P* < 0.0001) and mixed phenotype (LS mean change from baseline [95% CI]: −1.66 [–2.00, −1.31] vs −0.04 [–0.37, 0.29]; LS mean difference [95% CI]: −1.62 [–1.99, −1.25]; *P* < 0.0001). In addition, fibrostenotic subscore was lower with dupilumab vs placebo in patients with an inflammatory phenotype (LS mean change from baseline [95% CI]: 0.16 [–0.01, 0.33] vs 0.38 [0.21, 0.55]; −0.22 [–0.43, −0.01], *P* = 0.0438) and improved with dupilumab vs placebo in patients with a mixed phenotype (LS mean change from baseline [95% CI]: −0.95 [–1.21, –0.68] vs −0.29 [–0.54, –0.04]; −0.66 [–0.94, −0.38], *P* < 0.0001) (note that patients with inflammatory phenotype had 0 fibrostenotic subscore at baseline and so were unable to improve in this measure). These benefits were maintained at W52 in patients who received continuous dupilumab, and improvements were observed in those patients who switched to dupilumab at W24 (Figure 3).

**Figure 3.**
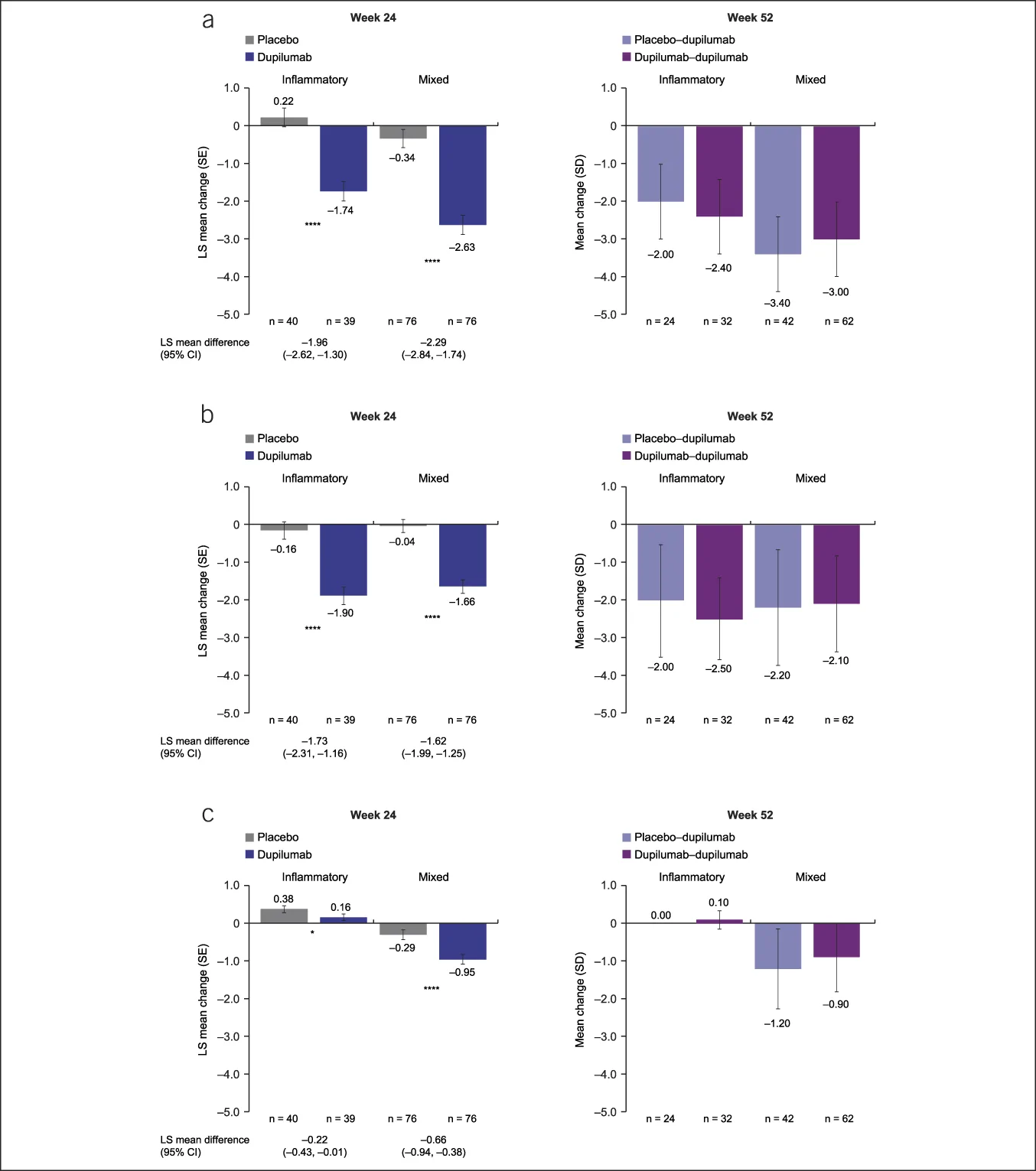
EREFS (**a**) total score, (**b**) inflammatory subscore, and (**c**) fibrostenotic subscore by inflammatory and mixed phenotypes at baseline. *Nominal *P* < 0.05, ****P < 0.0001 vs placebo. The CI with *P* value is based on the treatment difference (dupilumab group vs placebo) of the LS mean change using an ANCOVA model with covariate and the treatment, age group (≥12 to <18 vs ≥ 18 years), and PPI use at randomization (yes vs no) strata as fixed factors. ANCOVA, analysis of covariance; CI, confidence interval; EREFS, Eosinophilic Esophagitis Endoscopic Reference Score; LS, least squares; PPI, proton-pump inhibitor; SE, standard error.

At W24, 82.0% (95% CI 73.6, 88.6) vs 25.7% (17.6, 35.4) of patients in the dupilumab and placebo groups, respectively, achieved endoscopic remission (EREFS WOR total score ≤2) and 25.2% (17.5, 34.4) vs 5.9% (2.2, 12.5) achieved endoscopic normalization (EREFS WOR total score 0). In patients treated with continuous dupilumab, the proportion who achieved endoscopic remission and normalization at W52 (84.0% [75.3, 90.6] and 29.0% [20.4, 38.9], respectively) was similar to W24. In those patients who switched to dupilumab at W24, the proportion who achieved endoscopic remission and normalization at W52 (79.1% [67.4, 88.1] and 38.8% [27.1, 51.5], respectively) was increased compared with W24. The proportion of patients with each EREFS component score for those who had an EREFS WOR total score ≤2 at W24 and W52 are shown in Supplementary Figure S1 (see Supplementary Digital Content, https://links.lww.com/CTG/B588).

At W24, 45.0% (95% CI 35.6, 54.8) vs 7.9% (3.5, 15.0) of patients in the dupilumab and placebo groups, respectively, achieved an inflammatory subscore of 0 and 54.1% (44.3, 63.6) vs 35.3% (26.1, 45.4), respectively, achieved an EREFS fibrostenotic subscore of 0. At W52, the proportion of patients treated with continuous dupilumab who achieved an inflammatory subscore of 0 (48.0% [37.9, 58.2]) and fibrostenotic subscore of 0 (56.0% [45.7, 65.9]) was similar to W24. In those patients who switched to dupilumab at W24, the proportion who achieved an inflammatory subscore of 0 (50.7% [38.2, 63.2]) and fibrostenotic subscore of 0 (68.7% [56.2, 79.4]) at W52 was increased compared with W24.

Baseline covariates were assessed using univariate linear regression analysis, and the only baseline characteristics that had a significant effect on EREFS WOR total score at W24 were mixed profile and inflammatory profile. Mixed endoscopic phenotype seemed to be associated with greater improvement from baseline in EREFS WOR total score at W24, relative to patients with a nonmixed phenotype (for the purposes of the regression analysis, this was functionally equivalent to patients with an inflammatory phenotype), regardless of the treatment arm (estimated reduction for mixed vs nonmixed phenotype: −1.16 [95% CI –1.89, −0.44] in the dupilumab group, *P* = 0.0020 vs −0.94 [–1.73, −0.15] in the placebo group, *P* = 0.021), whereas inflammatory profile on EREFS WOR seemed to be associated with less improvement from baseline in the EREFS WOR total score at W24, relative to patients with a noninflammatory phenotype (functionally equivalent to patients with a mixed phenotype) in the placebo arm (estimated increase in inflammatory vs noninflammatory phenotype: 0.85 [0.05, 1.65], *P* = 0.0382). There was also a trend toward significance for patients with BMI ≥30 kg/m^2^ in the dupilumab arm (estimated increase in the dupilumab arm for BMI ≥30 kg/m^2^ vs < 25 kg/m^2^: 0.81 [–0.15, 1.77], *P* = 0.099) (see Supplementary Table S3, Supplementary Digital Content, https://links.lww.com/CTG/B588). The proportion of patients with each EREFS component score, for those with an inflammatory or mixed phenotype at W24 and W52, are shown in Supplementary Figure S2 (see Supplementary Digital Content, https://links.lww.com/CTG/B588).

Safety data for the LIBERTY EoE TREET have been previously reported (20,21).

## DISCUSSION

This post hoc analysis builds on the LIBERTY EoE TREET study that previously demonstrated that dupilumab 300 mg qw improves histologic outcomes and endoscopic features and alleviates symptoms of disease in adolescents and adults with EoE (20,21). In this article, we notably show that by W52, >80% of patients who received continuous dupilumab achieved endoscopic remission (EREFS WOR total score ≤2). Meeting this threshold is associated with increased likelihood of histologic and symptomatic response to treatment (22). In addition, nearly 30% had complete endoscopic normalization (EREFS WOR total score 0).

This post hoc analysis adopted the WOR method for analyzing EREFS in which only the worst affected esophageal region is scored for each component. A previous study by Ma et al (6) showed that this change improves the reliability and responsiveness of EREFS to treatment compared with the original, validated grading system. Importantly, WOR scoring also corresponds better to what is implemented in clinical practice than that in the original system (6). The results in this study demonstrated a larger relative difference between the dupilumab and placebo arms than was observed in the LIBERTY EoE TREET and EoE KIDS (NCT04394351) studies using the original grading system (20,23). This aligns with the increased responsiveness to treatment described for WOR scoring by Ma et al and suggests that future clinical studies including EREFS should consider using WOR scoring. However, our analysis was unable to determine whether EREFS by WOR scoring could detect changes in response to treatment earlier than the original EREFS grading system, due to the limited time points of endoscopies in TREET.

Clinicians previously believed that dilation was the only effective treatment of fibrotic EoE (4,7,24–28). It remains true that many patients with EoE still require dilation to address obstructive symptoms (3). However, in this study, fibrostenotic features were improved with dupilumab, with most improvement in rings. The modest improvements seen in strictures may have been limited by strictures being relatively rare at baseline because of the inclusion/exclusion criteria. Emerging real-world data in severely fibrostenotic EoE supports improvements in fibrostenosis with dupilumab; the study reported by Lee et al observed increases in stricture diameter with dupilumab. Although it did not assess whether these changes were clinically meaningful, they were statistically significant, and the global symptomatic response, as reported by patients, also improved (29). Long-term dupilumab treatment demonstrates sustained endoscopic improvements in both inflammation and fibrosis at W52. Notably, improvements in esophageal caliber and fibrostenosis have been associated with reduced symptoms, including dysphagia and food impaction and reduced need for esophageal dilations (26,30–32). The relationships between inflammatory and fibrostenotic histopathologic features of EoE and the inflammatory and fibrostenotic endoscopic features of EoE have been previously analyzed in TREET, with mixed results (33). The EREFS inflammation subscore was moderately correlated with the Eosinophilic Esophagitis Histology Scoring System (EoEHSS) grade and stage inflammatory subscore and EoEHSS grade and stage architectural (fibrostenotic) subscore. However, the EREFS fibrostenotic subscore was generally weakly correlated with the EoEHSS grade and stage architectural subscores (33). Aside from the lamina propria, which is often missing from esophageal biopsies, EoEHSS is limited to epithelial histologic features whereas the rings and strictures observed in endoscopy also involve subepithelial changes (4). Therefore, the lack of correlation between endoscopic and histologic fibrostenotic EREFS subscores in this study may suggest that visible fibrostenotic structural alterations more closely reflect changes in the subepithelium than changes in the epithelium. However, the specific contribution of subepithelial inflammation, smooth muscle hypertrophy, and subepithelial fibrosis to the fibrostenotic appearance observed on endoscopy remains uncertain (7,34,35). A larger, long-term study is required to investigate rates of fibrostenotic complications such as strictures in this population, and one such study (NCT06101095) is ongoing. Furthermore, complication rates in this study may have been reduced by both dupilumab treatment and exclusion of patients with narrow-caliber esophagus (esophageal strictures <10 mm).

At W24, improvements in inflammation subscores were greater than improvements in fibrostenotic subscores. Esophageal fibrostenosis is primarily driven by chronic type 2 inflammation (4), and esophageal fibrostenotic features seem to be more common with increasing age and disease duration (36,37). Fibrostenotic features may take longer to resolve than inflammatory features (4,7,24–28). Improvements in fibrostenotic aspects at W24 and W52 are consistent with previous reports of increased stricture diameter in a subset of patients with severe EoE treated with dupilumab (29). Although the improvements observed in fibrostenotic features in this study were relatively small, they were not a result of esophageal dilation as that procedure was only permitted as a rescue treatment.

Patients with a mixed inflammatory/fibrostenotic phenotype were on average ∼10 years older than patients with an inflammatory phenotype, although EoE disease duration was similar in both groups. Disease duration based on patient history is often unreliable because symptoms may be unrecognized or managed with long-standing adaptive behaviors (3). Previous findings suggest that a longer disease duration and delays in diagnosis, as well as increased age, are associated with an increased prevalence of fibrostenotic features in EoE (3,38,39). Based on these findings, the patients in the current cohort may have experienced substantial diagnostic delay.

This analysis has several strengths, including endoscopic outcomes collected systematically during a double-blind, randomized, placebo-controlled clinical trial. The most significant analysis limitation was its post hoc design. Patients with the most severe fibrostenotic type of EoE at baseline were excluded. An ongoing open-label study is evaluating dupilumab in patients with EoE with severe strictures (NCT06352073) (40,41). In addition, we did not assess a fibrostenotic-only phenotype as this was only present in a small number of patients, consistent with reports in the literature (39,42). Another limitation was the use of local scoring of EREFS WOR, which may increase inter-reader variability. However, observations in the placebo groups indicated that the variation between SDs at baseline and W24 in these measures was small, likely due to training provided to investigators on EREFS WOR, in an effort to mitigate variation. A possible limitation of EREFS is that, in our experience, it can be harder to see rings or strictures in some patients with active disease if edema is present. Therefore, it is possible that these fibrostenoic features may have been underreported at baseline in patients with edema. The interpretation of correlations between endoscopic observations and histology in the discussion is limited by the biopsy sites not matching the location of the observed endoscopic features in individual patients. Future studies that investigate the relationship between histopathology and endoscopy may wish to assess visual changes in specific, individual endoscopic features and sample tissue directly from the same location.

In conclusion, this post hoc analysis demonstrates that dupilumab treatment improves all endoscopic features (edema, rings, exudates, furrows, and strictures) of EoE vs placebo in most patients, regardless of baseline phenotype, by 52 weeks, using the clinically applicable EREFS WOR scoring system and newly proposed endoscopic response thresholds. These findings confirm the benefit of dupilumab treatment above and beyond improvements previously reported for the LIBERTY EoE TREET study.

## CONFLICTS OF INTEREST

**Guarantor of the article:** Evan S. Dellon, MD, MPH, FACG.

**Specific author contributions:** E.S.D.: conceptualization, investigation, methodology, data curation, writing—review & editing, approval of final draft. M.H.C.: investigation, methodology, data curation, writing—review and editing, approval of final draft. K.P.: investigation, methodology, data curation, writing—review and editing, approval of final draft. X.S.: formal analysis. B.P.R.: methodology, data curation, writing—review and editing, approval of final draft. M.L.: conceptualization, methodology, writing—original draft, approval of final draft. A.R.: conceptualization, methodology, writing—original draft; approval of final draft.

**Financial support:** Research sponsored by Sanofi and Regeneron Pharmaceuticals Inc. The study sponsors contributed to the study design, collection, analysis, and interpretation of the data and in the writing of the manuscript.

**Potential competing interests:** E.S.D. has received research funding from Adare/Ellodi, Allakos, Arena/Pfizer, AstraZeneca, Celgene/Receptos/Bristol Myers Squibb, Celldex, Dr Falk Pharma, Eupraxia, Ferring, GSK, Meritage, Miraca, Nutricia, Regeneron Pharmaceuticals Inc., Revolo, Sanofi, Shire/Takeda, and Uniquity; consultancy fees from AbbVie, Adare/Ellodi, Alfasigma, ALK, Allakos, Amgen, Apogee, Apollo, Aqilion, Arena/Pfizer, AstraZeneca, Bethanamist, Biocryst, Bryn, Calypso, Celgene/Receptos/Bristol Myers Squibb, Celldex, Domain, Dr Falk Pharma, EsoCap, Eupraxia, Ferring, GI Reviewers, GSK, Holoclara, Invea, Knightpoint, LucidDx, Nexstone Immunology/Uniquity, Nutricia, Parexel/Calyx, Phathom, Regeneron Pharmaceuticals Inc., Revolo, Robarts/Alimentiv, Roivant, Sanofi, Shire/Takeda, Target RWE, Third Harmonic Bio, and Upstream Bio; and educational grants from Allakos, Aqilion, Holoclara, and Invea. M.H.C. has received consultancy fees from Alimentiv, Arena/Pfizer, AstraZeneca, Calypso Biotech, EsoCap Biotech, GSK, Receptos/Celgene/Bristol Myers Squibb, Regeneron Pharmaceuticals Inc., Sanofi, and Shire/Takeda. K.P. holds stock and options in Nexeos Bio; has received speaker/consultancy/advisory fees from AGA, Allakos, Amgen, Anaptys Bio, Apogee, AstraZeneca, Bristol Myers Squibb, Celldex, GSK, Lucid, Peerview, Regeneron Pharmaceuticals Inc., Sanofi, Takeda, Uniquity, and WebMD; and receives unrestricted grant support fromAllakos and Chobani. X.S., B.P.R., and A.R. are employees of, and shareholders in, Regeneron Pharmaceuticals Inc. M.L. is an employee of Sanofi and may hold stock and/or stock options in the company.

**Trial registration:** ClinicalTrials.gov identifier: NCT03633617.

**Ethics approval:** The study was conducted in accordance with the Declaration of Helsinki, the International Conference on Harmonization Good Clinical Practice Guideline, and applicable regulatory requirements (Western Institutional Review Board number: 20181764). An independent data and safety monitoring committee conducted blinded monitoring of patient safety data. The local Institutional Review Board or Ethics Committee at each study center oversaw trial conduct and documentation. Pediatric patients provided assent according to the Ethics Committee (Institutional Review Board/Independent Ethics Committee)-approved standard practice for pediatric patients at each participating center.

**Patient and public involvement:** Patients and/or the public were not involved in the design, conduct, reporting, or dissemination plans of this research.

**Study Highlights**

**WHAT IS KNOWN**

✓ The eosinophilic esophagitis (EoE) Endoscopic Reference Score (EREFS) quantifies endoscopic disease severity based on findings of esophageal edema, rings, exudates, furrows, and strictures and provides a comprehensive platform to measure global esophageal endoscopic improvement.
✓ Systematic endoscopic monitoring is an important part of assessing treatment response and managing EoE because histopathology provides only an aspect of the disease.
✓ During the phase 3 LIBERTY EoE TREET study, weekly dupilumab led to improvements in EREFS total score (assessed by summing across regions), histology, and symptoms.

**WHAT IS NEW HERE**

✓ In this post hoc analysis, dupilumab improved all individual EREFS features of EoE as assessed by worst observed region.
✓ Endoscopic normalization was more frequent with dupilumab than placebo.
✓ Endoscopic features of EoE improved with dupilumab vs placebo, regardless of baseline study phenotype (inflammatory or mixed inflammatory/fibrostenotic).

## Supplementary Material

## ABBREVIATIONS:

BMI: body mass index
CI: confidence interval
EoE: eosinophilic esophagitis
EREFS: EoE Endoscopic Reference Score
EREFS WOR: EREFS based on worst observed regions
LS: least squares
qw: weekly
W: week
