# Supplementary material for: Dupilumab Improves Endoscopic Features in Adult and Adolescent Patients with Eosinophilic Esophagitis: Post Hoc Analysis of LIBERTY EoE TREET Study
Source: Clin Transl Gastroenterol. 2026 Aug 27;17(10):e01090. doi: 10.14309/ctg.0000000000001090 (PMC13694067; doi:10.14309/ctg.0000000000001090)
Supplement: Supplementary file 1 [file ct9-17-e01090-s001.docx]

Dupilumab Normalizes Endoscopic Features in Adult and Adolescent Patients With Eosinophilic Esophagitis: Post Hoc Analysis of LIBERTY EoE TREET Study

Evan S. Dellon, MD, MPH^1^, Margaret H. Collins, MD^2^, Kathryn Peterson, MD^3^, Xian Sun, PhD^4^, Bram P. Raphael, MD^4^, Margee Louisias, MD, MPH^5^ and Allen Radin, MD^4^

^1^Center for Esophageal Diseases and Swallowing, Division of Gastroenterology and Hepatology, University of North Carolina School of Medicine, Chapel Hill, North Carolina, USA; ^2^Division of Pathology and Laboratory Medicine, Department of Pediatrics, Cincinnati Children’s Hospital Medical Center, Department of Pathology & Laboratory Medicine, University of Cincinnati College of Medicine, Cincinnati, Ohio, USA; ^3^Division of Gastroenterology, University of Utah, Salt Lake City, Utah, USA; ^4^Regeneron Pharmaceuticals Inc., Tarrytown, New York, USA; ^5^Sanofi, Cambridge, Massachusetts, USA. **Correspondence:** Evan S. Dellon, MD, MPH. E-mail: [evan_dellon@med.unc.edu](mailto:evan_dellon@med.unc.edu).

**PLAIN LANGUAGE SUMMARY**

*•	Why was this study done?*

Eosinophilic esophagitis (EoE) is a progressive, chronic inflammatory disease affecting more and more people worldwide. Patients with EoE have symptoms such as difficulty swallowing and food impaction, which are caused by inflammation and remodeling of the esophagus. These changes can be viewed using an endoscope, and can be scored using the EoE-Endoscopic Reference Score (EREFS). EREFS is a validated diagnostic tool, however currently the minimal clinically important difference in EREFS is unknown. EREFS looks at three inflammatory signs and two remodeling signs in the esophagus. The presence and extent of these findings can be used to evaluate the severity of EoE. Dupilumab, a biologic therapy which blocks specific inflammatory pathways contributing to EoE, has been approved for EoE treatment in adults and adolescents. The authors of this study wanted to know if dupilumab was effective at reducing both esophageal inflammation and remodeling, as measured by EREFS. Furthermore, they evaluated methods to optimize the analysis and define responsiveness of endoscopic disease activity.

*•	What did the researchers do?*

LIBERTY EoE TREET was a large clinical study in which participants with EoE refractory to high-dose PPI therapy received dupilumab 300 mg weekly or placebo for 24 weeks, and then subsequently received dupilumab 300 mg weekly through 52 weeks. If they were on certain background treatments at the start of the trial, they continued taking them throughout. The primary endpoints were the proportion of patients having less than 6 eosinophils per high-power field on esophageal samples, and the change in Dysphagia Symptom Questionnaire score. Change in EREFS was a secondary endpoint. The participants underwent endoscopies to examine the esophagus before the trial began, and then at 24 weeks and 52 weeks. The EREFS score was calculated at each timepoint to determine the efficacy of dupilumab on esophageal inflammation and remodeling.

*•	What did the researchers find?*

The authors found that dupilumab was effective at improving esophageal inflammation and remodeling after 24 weeks of treatment when compared with patients taking a placebo. These benefits were maintained up to 52 weeks. The improvement in EREFS to dupilumab over placebo was increased by an analysis of worst observed region. Furthermore, dupilumab demonstrated responsiveness using thresholds defining endoscopic response.

*•	What do the findings mean?*

These findings show that dupilumab reduced inflammation and improved remodeling are important factors in the pathogenesis and clinical manifestations of EoE. Application of newer methods to analyze and define responsiveness of EREFS should help in future clinical trials in EoE.

**SUPPLEMENTARY METHODS**

**Assessments**

Eosinophilic Esophagitis Endoscopic Reference Score based on worst observed regions (EREFS WOR) was calculated by taking the highest score for each component from either the proximal or distal esophageal region, or, if region score was missing, the available one. Score ranges for the components were as follows: edema (absent/present [score range 0–1]), rings (absent/mild/moderate/severe [0–3]), exudates (absent/mild/severe [0–2]), furrows (absent/mild/severe [0–2]), and stricture (absent/present [0–1]), and were summed across proximal and distal regions for the previously published analyses. EREFS WOR inflammatory subscore comprised edema, exudates, and furrows (0–5), and the EREFS WOR fibrostenotic subscore comprised rings and stricture (0–4). While eligibility criteria excluded patients with strictures <10 mm or grade 3 rings, baseline features may have worsened over the study course.

| **Supplementary Table S1. Phenotype classification based on baseline EREFS component scores** | | |
| --- | --- | --- |
| **Phenotype** | **Baseline EREFS criteria** | |
|  | **Inflammatory component  scores** | **Fibrostenotic component scores** |
| Inflammatory only | Edema ≥1 OR Exudates ≥1 OR Furrows ≥1 | Rings = 0 AND Stricture = 0 |
| Mixed inflammatory/ fibrostenotic | Edema ≥1 OR Exudates ≥1 OR Furrows ≥1 | Rings ≥1 OR Stricture ≥1 |
| Fibrostenotic only | Edema = 0 AND Exudates = 0 AND Furrows = 0 | Rings ≥1 OR Stricture ≥1 |
| EREFS, Eosinophilic Esophagitis Endoscopic Reference Score. | | |

| Supplementary Table S2. Baseline demographics and characteristics by baseline endoscopic phenotype | | | | |
| --- | --- | --- | --- | --- |
|  | **Inflammatory** | | **Mixed inflammatory/fibrostenotic** | |
|  | **Placebo (n = 40)** | **Dupilumab (n = 39)** | **Placebo (n = 76)** | **Dupilumab (n = 76)** |
| Mean age, years (SD) | 20.4 (8.7) | 21.1 (12.8) | 32.0 (11.8) | 35.5 (12.7) |
| Sex, female, n (%) | 10 (25.0) | 12 (30.8) | 28 (36.8) | 29 (38.2) |
| Ethnicity, n (%) |  |  |  |  |
| Not Hispanic or Latino | 40 (100.0) | 37 (94.9) | 70 (92.1) | 69 (90.8) |
| Hispanic or Latino | 0 | 2 (5.1) | 6 (7.9) | 7 (9.2) |
| Race, n (%) |  |  |  |  |
| White | 33 (82.5) | 37 (94.9) | 75 (98.7) | 70 (92.1) |
| Black or African American | 4 (10.0) | 1 (2.6) | 0 | 2 (2.6) |
| Asian | 0 | 0 | 0 | 2 (2.6) |
| Other/Not reported | 3 (7.5) | 1 (2.6) | 1 (1.3) | 2 (2.6) |
| History of allergic rhinitis, n (%) | 29 (72.5) | 28 (71.8) | 45 (59.2) | 42 (55.3) |
| History of food allergy, n (%) | 22 (55.0) | 25 (64.1) | 36 (47.4) | 35 (46.1) |
| History of asthma, n (%) | 16 (40.0) | 21 (53.8) | 27 (35.5) | 27 (35.5) |
| History of atopic dermatitis, n (%) | 11 (27.5) | 10 (25.6) | 19 (25.0) | 14 (18.4) |
| History of prior swallowed topical corticosteroid use for EoE, n (%) | 28 (70.0) | 28 (71.8) | 58 (76.3) | 52 (68.4) |
| History of esophageal dilation, n (%) | 6 (15.0) | 6 (15.4) | 43 (56.6) | 36 (47.4) |
| PPI use at randomization, n (%) | 29 (72.5) | 21 (53.8) | 55 (72.4) | 59 (77.6) |
| On a food elimination diet at screening, n (%) | 18 (45.0) | 24 (61.5) | 27 (35.5) | 21 (27.6) |
| Duration of EoE, years, mean (SD) | 4.4 (4.6) | 5.9 (4.5) | 5.1 (4.4) | 5.5 (4.6) |
| PEC in 3 regions, mean eos/hpf (SD) | 76.3 (45.8) | 86.7 (46.6) | 95.4 (45.4) | 89.6 (43.9) |
| DSQ score, mean (SD) | 36.5 (10.5) | 40.4 (10.8) | 35.5 (11.4) | 34.8 (11.6) |
| EREFS WOR total score, mean (SD) | 3.0 (1.1) | 3.3 (1.0) | 4.6 (1.5) | 4.5 (1.4) |
| EoEHSS grade total score, mean (SD) | 1.2 (0.5) | 1.3 (0.4) | 1.3 (0.4) | 1.3 (0.4) |
| EoEHSS stage total score, mean (SD) | 1.2 (0.4) | 1.4 (0.3) | 1.3 (0.3) | 1.3 (0.3) |
| DSQ, Dysphagia Symptom Questionnaire; EoE, eosinophilic esophagitis; EoEHSS, EoE Histology Scoring System; eos/hpf, eosinophils per high-power field; EREFS, EoE Endoscopic Reference Score; EREFS WOR, EREFS based on worst observed regions; PEC, peak esophageal intraepithelial eosinophil count; PPI, proton pump inhibitor. | | | | |

| **Supplementary Table S3. Univariate linear regression analysis of EREFS total score at week 24 by baseline covariate** | | | | |
| --- | --- | --- | --- | --- |
| **Baseline covariate** | **Dupilumab (n = 118)** | | **Placebo (n = 122)** | |
|  | **Estimate (95% CI)** | **Pr >\|t\|** | **Estimate (95% CI)** | **Pr >\|t\|** |
| Weight ≥60 kg vs <60 kg (Reference) | 0.02 (–0.84, 0.89) | 0.96 | 0.02 (–0.97, 1.01) | 0.96 |
| BMI ≥25—<30 kg/m^2^ vs <25 kg/m^2^ (Reference) | 0.06 (–0.78, 0.89) | 0.89 | –0.63 (–1.54, 0.28) | 0.17 |
| BMI ≥30 kg/m^2^ vs <25 kg/m^2^ (Reference) | 0.81 (–0.15, 1.77) | 0.10 | –0.28 (–1.29, 0.74) | 0.59 |
| Duration of EoE ≥5 years vs <5 years (Reference) | 0.06 (–0.68, 0.79) | 0.88 | 0.11 (–0.69, 0.90) | 0.79 |
| History of atopic dermatitis vs no history of atopic dermatitis (Reference) | –0.53 (–1.41, 0.36) | 0.24 | –0.33 (–1.19, 0.53) | 0.45 |
| Food elimination at screening vs no food elimination at screening (Reference) | 0.69 (–0.05, 1.42) | 0.07 | 0.43 (–0.37, 1.22) | 0.29 |
| Treated with PPI at randomization per IWRS vs not treated with PPI at randomization per IWRS (Reference) | –0.27 (–1.04, 0.50) | 0.49 | –0.57 (–1.40, 0.26) | 0.18 |
| Treated with PPI at randomization per EDC vs not treated with PPI at randomization per EDC (Reference) | –0.04 (–0.83, 0.75) | 0.93 | –0.66 (–1.51, 0.20) | 0.13 |
| Patients with an inflammatory phenotype vs patients without an inflammatory phenotype (Reference) | 0.71 (–0.05, 1.48) | 0.07 | 0.85 (0.05, 1.65) | 0.04 |
| Patients with a mixed phenotype vs patients without a mixed phenotype (Reference) | –1.16 (–1.89, –0.44) | <0.01 | –0.94 (–1.73, –0.15) | 0.02 |
| BMI, body mass index; CI, confidence interval; EDC, electronic data capture; EoE, eosinophilic esophagitis; EREFS, EoE Endoscopic Reference Score; IWRS, interactive web response system; PPI, proton pump inhibitor; Pr >\|t\|, two-sided p value from the t test of H0: the regression coefficient equals 0. Positive estimates indicate higher EREFS versus the reference category; negative estimates indicate lower EREFS. Models were fit separately within each treatment arm. | | | | |

**Supplementary Figure S1.** The proportion of patients with each EREFS component score for those who had an EREFS WOR total score ≤2 at week 24 and week 52. N, number of patients with non-missing values at each visit. EREFS, Eosinophilic Esophagitis Endoscopic Reference Score; EREFS WOR, EREFS based on worst observed regions.

**Supplementary Figure S2.** The proportion of patients with each EREFS component score for those with an (A) inflammatory or (B) mixed inflammatory/fibrostenotic phenotype at week 24 and week 52. N, number of patients with non-missing values at each visit. EREFS, Eosinophilic Esophagitis Endoscopic Reference Score.
